# Systemic inflammation and mental health: A J-shaped dose-response pattern in depression based on NHANES 2005 to 2018

**DOI:** 10.1097/MD.0000000000049348

**Published:** 2026-07-03

**Authors:** Guanwen Zhu, Jiawen Liu, Sijia Wang, Long Wang

**Affiliations:** a Graduate School, Heilongjiang University of Chinese Medicine, Harbin, Heilongjiang, China; b First Affiliated Hospital, Heilongjiang University of Chinese Medicine, Harbin, Heilongjiang, China.

**Keywords:** cross-sectional study, depression, NHANES database, PIV index, SII index, SIRI index

## Abstract

Inflammation is increasingly being recognized as a central factor in the development of depression, a common and debilitating condition. However, the relationship between depression and systemic inflammation indices – systemic immune-inflammation index (SII), pan-immune-inflammatory value (PIV), and systemic inflammatory response index (SIRI) – is not fully understood. In this cross-sectional study, data from the National Health and Nutrition Examination Survey 2005 to 2018, including 22,936 adults in the United States, were analyzed using multivariate logistic regression, smoothed curve fitting, and threshold and subgroup analyses to examine the associations between SII, PIV, SIRI, and depression, as well as their potential nonlinear characteristics. The analysis revealed that a 1-unit increase in Ln(SII), Ln(PIV), and Ln(SIRI) was associated with 18.2%, 14%, and 9.5% higher odds of prevalent depression, respectively. Nonlinear “J-shaped” relationships were observed for all the 3 indices. The threshold effect analysis identified significantly higher odds for Ln(SII) > 6.187, Ln(PIV) > 5.581, and Ln(SIRI) > 0.95. The subgroup analysis showed that smoking, diabetes, and liver disease significantly modified these associations. SII, PIV, and SIRI are closely linked to depressive symptoms, suggesting that they could serve as accessible inflammatory correlates of depression. Longitudinal research is required to test their prognostic utility and unravel their underlying biology.

## 1. Introduction

Depression is a critical public health issue in the 21st century. This mood disorder is chiefly marked by ongoing feelings of sadness and a diminished capacity for enjoyment or interest, frequently coupled with cognitive difficulties and physical complaints.^[1]^ As of 2019, over 320 million individuals have been affected by depression globally, accounting for 4.3% of the world’s population. Its impact, assessed in terms of disability-adjusted life years, exceeds that of conventional chronic illnesses, such as diabetes, positioning depression as the foremost contributor to disability among nonfatal conditions.^[2]^ In addition to its substantial impact on personal well-being, depression places a considerable strain on familial and societal structures.

Immune cells and inflammatory agents such as interleukin-1 beta (IL-1β), IL-6, tumor necrosis factor-α, and C-reactive protein have long been associated with biological pathways contributing to depression. These indicators influence the development and progression of depression by modulating neurotransmitter activity, neural plasticity, and inflammatory processes in the brain.^[3,4]^ In recent years, the systemic immune-inflammation index (SII), pan-immune-inflammatory value (PIV), and systemic inflammatory response index (SIRI) have emerged as promising research avenues for depression because of their ability to reflect the dynamic balance of neutrophils, lymphocytes, monocytes, and platelets.^[5,6]^ The SII, in particular, serves as a comprehensive indicator of immune-inflammatory status and has been found to correlate significantly with depressive symptoms. It can affect neuroinflammatory processes in depression by regulating the interplay between peripheral immune cells and the central nervous system.^[7,8]^ Although the predictive significance of PIV and SIRI has been investigated in disorders such as cardiovascular and cerebrovascular conditions, their role in depression remains underexplored. Based on these findings, we hypothesize that systemic inflammatory markers may provide new insights into the multifaceted pathophysiological mechanisms underlying depression.^[9–11]^

Therefore, the present study aimed to examine the associations between 3 systemic inflammation-based indices – SII, PIV, and SIRI – and depression in a nationally representative sample of United States (U.S.) adults. Using data from the National Health and Nutrition Examination Survey (NHANES), we also explored the potential nonlinear nature of these relationships and examined whether key demographic and clinical factors, such as smoking, diabetes, and liver disease, modify these associations. This study seeks to inform future public health strategies by identifying potential inflammation-related targets in depression.

## 2. Methods

### 2.1. Study design and population

The data for this study were sourced from NHANES, conducted by the National Center for Health Statistics (NCHS) from 2005 to 2018. NHANES is a unique, ongoing cross-sectional survey that assesses the health and nutritional status of the noninstitutionalized civilian population in the United States. It combines interviews and physical examinations, with ethical approval for data collection protocols provided by the NCHS Research Ethics Review Board. Written informed consent was obtained from all participants. For additional details, please refer to the official website https://www.cdc.gov/nchs/nhanes/.

**Figure 1.**
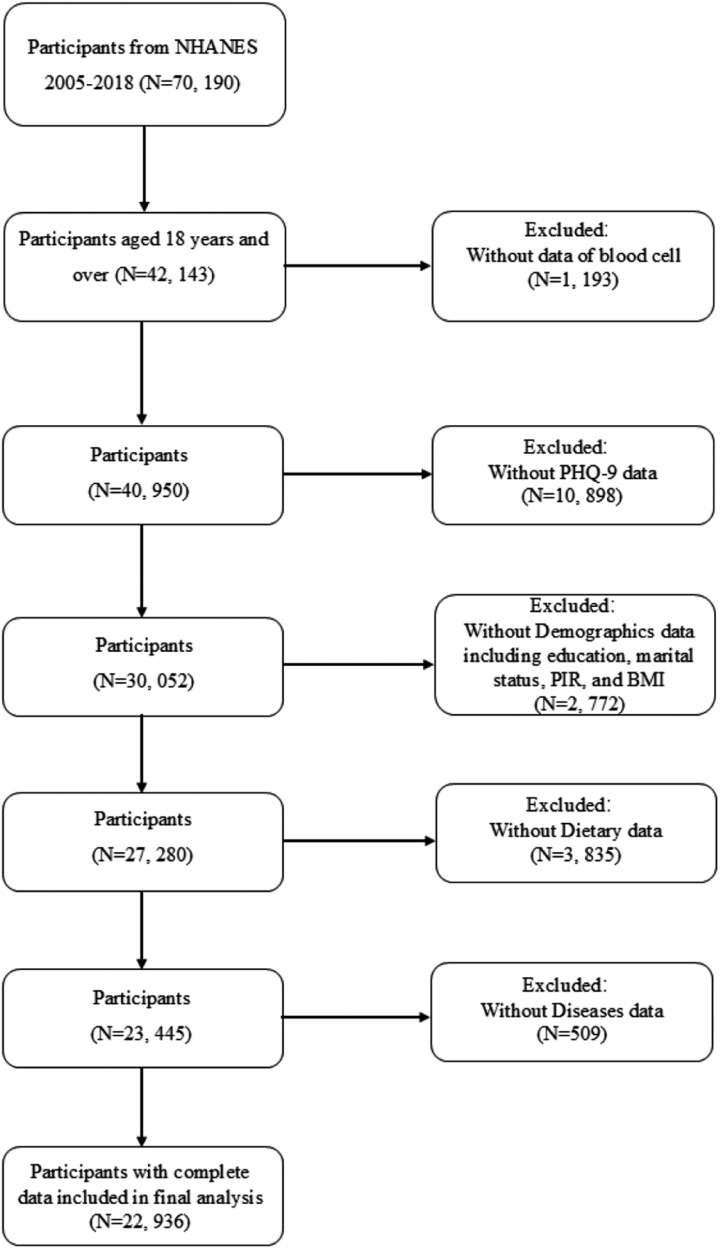
Diagram of participant enrollment process. BMI = body mass index, NHANES = National Health and Nutrition Examination Survey, PHQ-9 = 9-item Patient Health Questionnaire, PIR = poverty impact ratio.

In this study, we included all NHANES participants from 2005 to 2018 (n = 70,190). Individuals under 18 years of age (n = 28,047), participants lacking complete blood count data (n = 1193), those without depression diagnostic information (n = 10,898), and those with missing covariate details (n = 7116) were excluded. As the proportion of missing covariate data was very small, we performed a complete-case analysis after excluding participants with missing values. Ultimately, 22,936 participants were analyzed (Fig. 1).

All regression models incorporated survey weights to account for the complex sampling design of the NHANES. Because complete blood count assays were standardized across survey cycles by NCHS, and prior NHANES-based studies have not reported meaningful cycle-specific differences in these indices, survey cycle fixed effects or trend terms were not included in the primary models.^[9,12]^

### 2.2. Systemic inflammation markers and group classification

SII = (platelet count × neutrophil count)/lymphocyte count, PIV = (platelet count × neutrophil count × monocyte count)/lymphocyte count, and SIRI = (neutrophil count × monocyte count)/lymphocyte count.

All blood cell counts were obtained from the NHANES complete blood count data files and were expressed in units of × 10^3^ cells/μL before transformation. Laboratory methods for complete blood counts were standardized across survey cycles by NCHS, and no instrument-specific adjustments were required. Natural logarithm transformations were applied to address the skewed distributions of these indices. For comparative analysis, participants were stratified into quartile-based categories according to the Ln-transformed values of the SII, PIV, and SIRI.^[9]^

### 2.3. Depression diagnosis

The severity of depressive symptoms was assessed using the 9-item Patient Health Questionnaire (PHQ-9). A total score of ≥10 (out of 27) was used to define clinically relevant depressive symptoms, consistent with validation studies demonstrating high sensitivity and specificity for major depressive disorders in primary care settings.^[13]^ This cutoff is widely applied in epidemiological research and aligns with our study objective of identifying clinically relevant cases. Missing PHQ-9 items were handled using a complete case approach: PHQ-9 total scores were calculated only for participants with non-missing responses on all 9 items. Participants with missing data on any PHQ-9 item were excluded from the analyses involving depressive symptoms.

### 2.4. Other covariates

Information on the participants’ sociodemographic characteristics, lifestyle factors, and diseases affecting depression was collected. Sociodemographic traits included age, sex, race, educational level, poverty impact ratio (PIR), and marital status. Behavioral determinants included body mass index (BMI), which was derived from standardized height and weight measurements. Serum cotinine levels were measured to assess smoking status, categorizing participants as light, moderate, or heavy smokers (<0.011, 0.011 to <0.11, and ≥0.11 ng/mL).^[14]^ Alcohol consumption was evaluated based on the frequency of alcohol consumption over the previous 12 months. Clinical comorbidities related to depression, such as hypertension, diabetes, stroke, arthritis, heart failure, and other chronic diseases, were assessed using questionnaires. Due to data limitations, information on anti-inflammatory or psychotropic medication use, recent infections, and autoimmune disorders was not uniformly available across all survey cycles and was therefore not included as a covariate.

### 2.5. Statistical analysis

To account for NHANES’s complex survey design of NHANES, we applied sampling weights (WTMEC2YR), strata (SDMVSTRA), and primary sampling units (SDMVPSU). For the pooled cycles, the weights were divided by the number of cycles. Design-based variance estimation was employed to account for the stratified, clustered sampling design. Data were analyzed using R version 4.2.3 (R Core Team) (package survey, function svyglm) and EmpowerStats 2.0 (X & Y Solutions, Inc.) (α = 0.05). Baseline characteristics were compared according to depression status using weighted *t* tests (means ± SE) and chi-square tests (frequencies, %). Logistic regression was used to assess associations between inflammatory markers and depression: Model 1 (unadjusted), Model 2 (adjusted for age, sex, and race), and Model 3 (further adjusted for education, marital status, PIR, BMI, smoking, and alcohol). Given that SII, PIV, and SIRI are composite indices that share common blood cell components (neutrophils, lymphocytes, and platelets), they are strongly correlated with 1 another. To avoid multicollinearity and ensure a clear interpretation of the results, each biomarker was analyzed in a separate regression model rather than being included simultaneously.^[9,12]^ Smooth curve fitting and threshold analysis were used to examine Ln(SII), Ln(PIV), and Ln(SIRI). Correlation analysis assessed biomarker interrelations, and subgroup analyses explored consistency and effect modification (details in Information S1, Supplemental Digital Content 1).

## 3. Results

### 3.1. Characteristics of the included participants

After excluding participants who lacked routine blood tests or depression data, 22,936 individuals were analyzed. Participants were divided into 2 groups based on their depression status. The depressed group was younger, mostly female, less educated, unmarried, had a higher BMI and lower PIR, and were more often non-Hispanic White, with unhealthy lifestyle habits and more comorbidities. Notably, the Ln(SII), Ln(PIV), and Ln(SIRI) levels were significantly higher in this group (Table 1).

**Table 1:** Weighted comparison in basic characteristics.

	Overall	Depression		*P* value
Characteristics		Without	With	
	n = 22936	n = 21202	n = 1734	
Age	51.281 ± 16.072	51.535 ± 16.147	48.170 ± 14.780	<.001
Lymphocyte, 1000 cells/μL	2.180 ± 2.713	2.173 ± 2.814	2.265 ± 0.759	.177
Segmented neutrophils, 1000 cells/μL	4.295 ± 1.834	4.259 ± 1.816	4.725 ± 1.991	<.001
Eosinophilic lymphocytes, 1000 cells/μL	0.206 ± 0.179	0.205 ± 0.181	0.214 ± 0.163	.042
Basophilic lymphocytes, 1000 cells/μL	0.047 ± 0.066	0.046 ± 0.067	0.051 ± 0.056	.003
Platelet, 1000 cells/μL	246.998 ± 66.821	246.053 ± 66.279	258.561 ± 72.142	<.001
SII	544.058 ± 388.982	540.116 ± 389.277	592.258 ± 382.189	<.001
Ln (SII)	6.147 ± 0.548	6.141 ± 0.546	6.224 ± 0.567	<.001
PIV	314.970 ± 298.765	312.070 ± 298.575	350.431 ± 298.895	<.001
Ln (PIV)	5.514 ± 0.684	5.506 ± 0.681	5.609 ± 0.706	<.001
SIRI	1.257 ± 0.921	1.251 ± 0.916	1.334 ± 0.979	<.001
Ln (SIRI)	0.040 ± 0.607	0.036 ± 0.606	0.093 ± 0.618	<.001
Gender				<.001
Male	12,072 (52.633%)	11,406 (53.797%)	666 (38.408%)	
Female	10,864 (47.367%)	9796 (46.203%)	1068 (61.592%)	
Education level (%)				<.001
<9th	1926 (8.397%)	1726 (8.141%)	200 (11.534%)	
9–11th	3076 (13.411%)	2723 (12.843%)	353 (20.358%)	
High school	5249 (22.885%)	4812 (22.696%)	437 (25.202%)	
Some college	6848 (29.857%)	6311 (29.766%)	537 (30.969%)	
College graduate	5837 (25.449%)	5630 (26.554%)	207 (11.938%)	
Marry (%)				<.001
Unmarried	10,182 (44.393%)	9068 (42.770%)	1114 (64.245%)	
Married	12,754 (55.607%)	12,134 (57.230%)	620 (35.755%)	
PIR				<.001
<1	4072 (17.754%)	3448 (16.263%)	624 (35.986%)	
≥1, <3	9440 (41.158%)	8643 (40.765%)	797 (45.963%)	
≥3	9424 (41.088%)	9111 (42.972%)	313 (18.051%)	
BMI (kg/m^2^)				<.001
<25	6099 (26.591%)	5699 (26.880%)	400 (23.068%)	
25–30	7766 (33.859%)	7308 (34.468%)	458 (26.413%)	
≥30	9071 (39.549%)	8195 (38.652%)	876 (50.519%)	
Smoking(%)				
Low	7379 (32.172%)	7046 (33.233%)	333 (19.204%)	
Medium	7907 (34.474%)	7424 (35.016%)	483 (27.855%)	
High	7650 (33.354%)	6732 (31.752%)	918 (52.941%)	
Drinking (%)				<.001
Low	4029 (17.566%)	3476 (16.395%)	553 (31.892%)	
High	18,907 (82.434%)	17,726 (83.605%)	1181 (68.108%)	
Hypertension (%)				<.001
Yes	8646 (37.696%)	7847 (37.011%)	799 (46.078%)	
No	14,290 (62.304%)	13,355 (62.989%)	935 (53.922%)	
Asthma (%)				<.001
Yes	3316 (14.458%)	2887 (13.617%)	429 (24.740%)	
No	19,620 (85.542%)	18,315 (86.383%)	1305 (75.260%)	
Arthritis (%)				<.001
Yes	6673 (29.094%)	5918 (27.912%)	755 (43.541%)	
No	16,263 (70.906%)	15,284 (72.088%)	979 (56.459%)	
Congestive cardiac failure (%)				<.001
Yes	752 (3.279%)	640 (3.019%)	112 (6.459%)	
No	22,184 (96.721%)	20,562 (96.981%)	1622 (93.541%)	
Coronary heart disease (%)				<.001
Yes	989 (4.312%)	886 (4.179%)	103 (5.940%)	
No	21,947 (95.688%)	20,316 (95.821%)	1631 (94.060%)	
Angina pectoris (%)				<.001
Yes	625 (2.725%)	521 (2.457%)	104 (5.998%)	
No	22,311 (97.275%)	20,681 (97.543%)	1630 (94.002%)	
Acute infarction (%)				<.001
Yes	1031 (4.495%)	894 (4.217%)	137 (7.901%)	
No	21,905 (95.505%)	20,308 (95.783%)	1597 (92.099%)	
Stroke (%)				<.001
Yes	859 (3.745%)	720 (3.396%)	139 (8.016%)	
No	22,077 (96.255%)	20,482 (96.604%)	1595 (91.984%)	
Pulmonary emphysema (%)				<.001
Yes	541 (2.359%)	446 (2.104%)	95 (5.479%)	
No	22,395 (97.641%)	20,756 (97.896%)	1639 (94.521%)	
Chronic bronchitis (%)				<.001
Yes	1442 (6.287%)	1186 (5.594%)	256 (14.764%)	
No	21,494 (93.713%)	20,016 (94.406%)	1478 (85.236%)	
Liver disease (%)				<.001
Yes	986 (4.299%)	825 (3.891%)	161 (9.285%)	
No	21,950 (95.701%)	20,377 (96.109%)	1573 (90.715%)	
Malignant tumor (%)				<.001
Yes	2401 (10.468%)	2128 (10.037%)	273 (15.744%)	
No	20,535 (89.532%)	19,074 (89.963%)	1461 (84.256%)	
Thyroid disease (%)				.046
Yes	2364 (10.307%)	2161 (10.192%)	203 (11.707%)	
No	20,572 (89.693%)	19,041 (89.808%)	1531 (88.293%)	
Diabetes (%)				<.001
Yes	2983 (13.006%)	2671 (12.598%)	312 (17.993%)	
No	19,388 (84.531%)	18,028 (85.030%)	1360 (78.431%)	
Prediabetes	565 (2.463%)	503 (2.372%)	62 (3.576%)	

BMI = body mass index, PIR = poverty impact ratio, PIV = pan-immune-inflammatory value, SII = systemic immune-inflammation index, SIRI = systemic inflammatory response index.

### 3.2. Association of SII, PIV, and SIRI with depression

In fully adjusted Model 3 of the logistic regression analysis, a 1-unit elevation in Ln(SII) was linked to an 18.2% greater likelihood of depression (odds ratio [OR] = 1.182, 95% confidence interval [CI] = 1.075–1.300, *P* < .001). Similarly, Ln(PIV) showed a significant positive relationship with the prevalence of depression (OR = 1.140, 95% CI = 1.056–1.231, *P* < .001). Higher levels of Ln(PIV) were strongly associated with higher odds of depression, with the Q4 quartile showing 27.6% higher odds than Q1 (OR = 1.276, 95% CI = 1.102–1.478, *P* = .001). Furthermore, elevated SIRI levels were significantly associated with increased odds of depression (OR = 1.095, 95% CI = 1.040–1.152, *P* < .001). Individuals in the highest quartile (Q4) of Ln(SIRI) exhibited 26.6% increased odds of prevalent depression relative to the lowest quartile (Q1) (OR = 1.266, 95% CI = 1.094–1.467, *P* = .002) (Table 2).

**Table 2:** Survey-weighted logistic regression examining the association of SII, PIV, SIRI with the prevalence of depression among individuals from NHANES 2005 to 2018.

	Model 1	Model 2	Model 3
SII	1.000 (1.000, 1.000)*	1.000 (1.000, 1.000)*	1.000 (1.000, 1.000)*
Ln(SII)	1.320 (1.207, 1.445)*	1.280 (1.166, 1.405)*	1.182 (1.075, 1.300)*
Ln(SII) quartile			
Q1	Ref.	Ref.	Ref.
Q2	1.092 (0.944, 1.264)	1.064 (0.918, 1.233)	1.081 (0.929, 1.259)
Q3	1.098 (0.949, 1.270)	1.044 (0.900, 1.210)	0.991 (0.851, 1.155)
Q4	1.522 (1.327, 1.745)*	1.432 (1.244, 1.648)*	1.284 (1.111, 1.485)*
PIV	1.000 (1.000, 1.000)*	1.000 (1.000, 1.001)*	1.000 (1.000, 1.000)*
Ln(PIV)	1.246 (1.160, 1.339)*	1.276 (1.185, 1.374)*	1.140 (1.056, 1.231)*
Ln(PIV) quartile			
Q1	Ref.	Ref.	Ref.
Q2	1.046 (0.903, 1.212)	1.055 (0.909, 1.224)	1.028 (0.882, 1.198)
Q3	1.183 (1.025, 1.365)*	1.191 (1.029, 1.378)*	1.068 (0.918, 1.242)
Q4	1.482 (1.292, 1.701)*	1.539 (1.336, 1.773)*	1.276 (1.102, 1.478)*
SIRI	1.087 (1.039, 1.138)*	1.148 (1.097, 1.201)*	1.095 (1.040, 1.152)*
Ln(SIRI)	1.169 (1.078, 1.267)*	1.310 (1.204, 1.426)*	1.162 (1.064, 1.269)*
Ln(SIRI) quartile			
Q1	Ref.	Ref.	Ref.
Q2	0.924 (0.799, 1.068)	0.968 (0.835, 1.121)	0.936 (0.804, 1.089)
Q3	1.035 (0.899, 1.192)	1.133 (0.980, 1.310)	0.995 (0.857, 1.156)
Q4	1.294 (1.131, 1.481)*	1.535 (1.333, 1.768)*	1.266 (1.094, 1.467)*

Model 1 did not adjust for any covariates. Model 2 adjusted for age, gender, and race. Model 3 adjusted for age, gender, race, education level, marital status, PIR, BMI, smoking, and drinking. Data marked with an asterisk (*) indicate significant differences (*P* < .05).

BMI = body mass index, PIR = poverty impact ratio, PIV = pan-immune-inflammatory value, SII = systemic immune-inflammation index, SIRI = systemic inflammatory response index.

### 3.3. Smooth curve fitting and threshold effects

**Figure 2.**
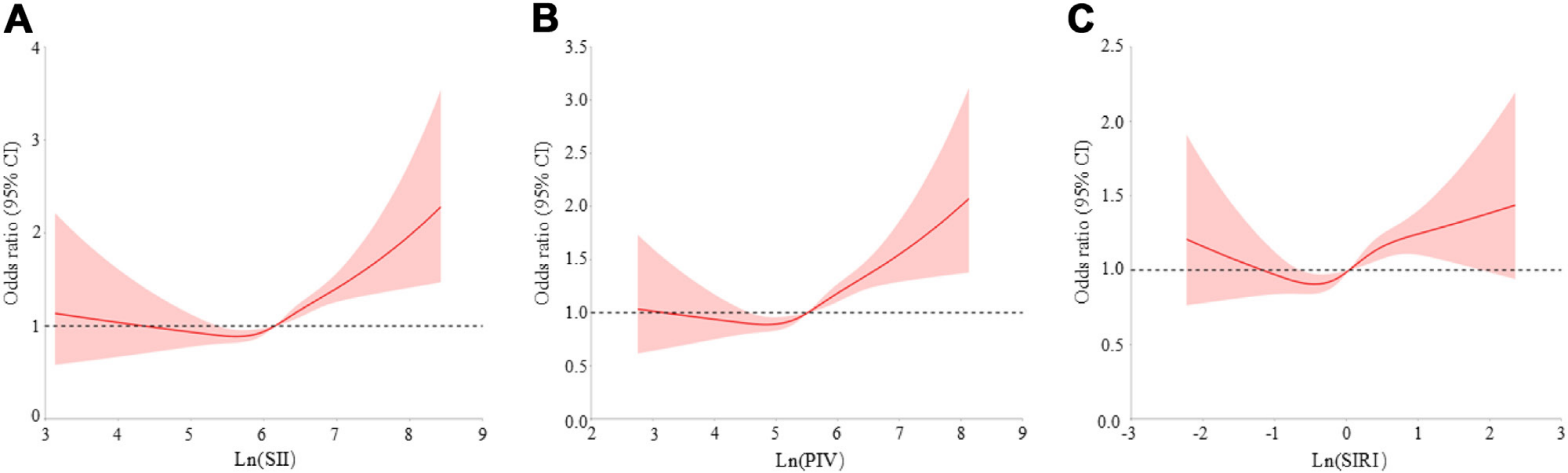
Associations of Ln(SII) (A), Ln(PIV) (B), and Ln(SIRI) (C) with odds of prevalent depression (RCS models). Models adjusted for age, sex, race, education, marital status, PIR, BMI, smoking, and alcohol. Red line: estimated OR; Shaded area: 95% CI. Estimates are survey-weighted. BMI = body mass index, CI = confidence interval, OR = odds ratio, PIR = poverty impact ratio, PIV = pan-immune-inflammatory value, RCS = restricted cubic splines, SII = systemic immune-inflammation index, SIRI = systemic inflammatory response index.

To further explore the associations of SII, PIV, and SIRI with depression, we fitted restricted cubic spline models with 4 knots placed at equally spaced percentiles of each biomarker distribution following standard recommendations^[15]^ (Table 3, Fig. 2). Global likelihood ratio tests indicated significant nonlinearity for Ln(SII) (*P* = .012), Ln(PIV) (*P *= .037), and Ln(SIRI) (*P* = .002). The spline curves revealed J-shaped relationships: odds ratios remained close to or slightly below 1 at lower biomarker levels, reached a nadir, and then rose steeply at higher levels. Segmented regression analyses identified inflection points at Ln(SII) = 6.187, Ln(PIV) = 5.581, and Ln(SIRI) = 0.950, respectively. Above these thresholds, the odds of prevalent depression increased markedly, with adjusted ORs of 1.561 (95% CI = 1.337–1.822, *P* < .001) for the SII, 1.401 (95% CI = 1.231–1.594, *P* < .001) for the PIV, and 1.086 (95% CI = 1.031–1.144, *P* = .002) for the SIRI. These findings suggest potential threshold effects; however, as they are data-driven, they should be considered exploratory and interpreted with caution.

**Table 3:** Analysis of threshold effect.

Ln(SII)	Adjusted OR (95% CI), *P* value	Ln(PIV)	Adjusted OR (95% CI), *P* value	Ln(SIRI)	Adjusted OR (95% CI), *P* value
Fold points (*K*)	6.187	Fold points (*K*)	5.581	Fold points (*K*)	0.950
Ln(SII) ≤ 6.187	1.094 (0.925–1.293) .2936	Ln(PIV) ≤ 5.581	1.100 (0.961–1.260) .1654	Ln(SIRI) ≤ 0.950	1.104 (0.845–1.443) .4689
Ln(SII) > 6.187	1.561 (1.337–1.822) < .0001	Ln(PIV) > 5.581	1.401 (1.231–1.594) <.0001	Ln(SIRI) > 0.950	1.086 (1.031–1.144) .0019
Effect size difference of 2 vs 1	1.427 (1.087–1.873) .0103	Effect size difference of 2 vs 1	1.273 (1.017–1.594) .0355	Effect size difference of 2 vs 1	0.983 (0.737–1.313) .9098
Equation predicted values at break points	−2.580 (−2.658 to −2.503)	Equation predicted values at break points	−2.564 (−2.643 to −2.485)	Equation predicted values at break points	−2.529 (−2.599 to −2.460)
Log likelihood ratio tests	0.012	Log likelihood ratio tests	0.037	Log likelihood ratio tests	0.910

CI = confidence interval, OR = odds ratio, PIV = pan-immune-inflammatory value, SII = systemic immune-inflammation index, SIRI = systemic inflammatory response index.

### 3.4. Stratified analysis

**Figure 3.**
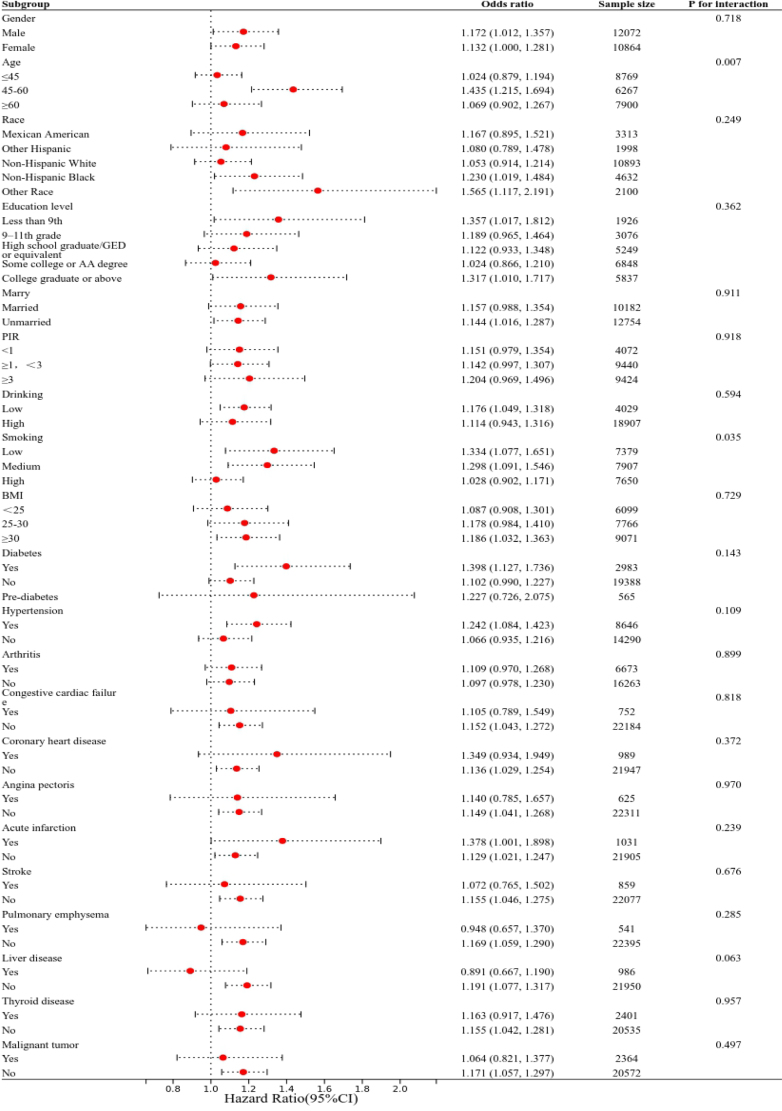
Subgroup analysis of the association between Ln(SII) and depression. Models adjusted for age, sex, race, education, marital status, PIR, BMI, smoking, and alcohol (stratification variables excluded). Estimates are survey-weighted. BMI = body mass index, CI = confidence interval, GED = General Educational Development, PIR = poverty impact ratio, SII = systemic immune-inflammation index.

**Figure 4.**
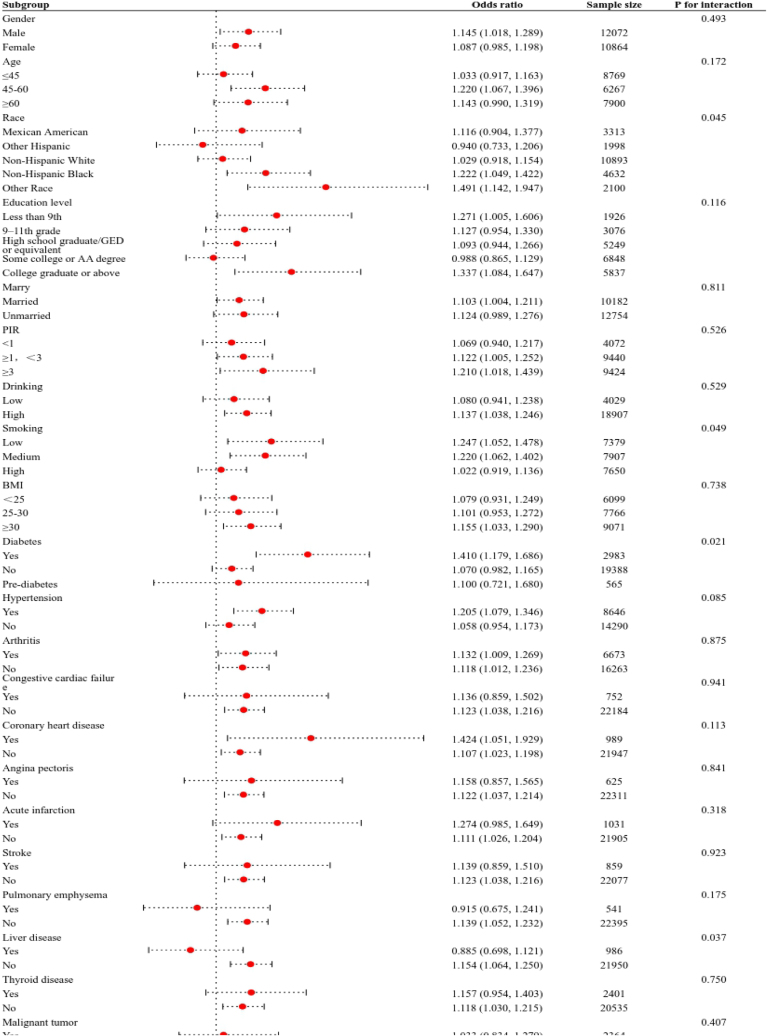
Subgroup analysis of the association between Ln(PIV) and depression. Models adjusted for age, sex, race, education, marital status, PIR, BMI, smoking, and alcohol (stratification variables excluded). Estimates are survey-weighted. BMI = body mass index, CI = confidence interval, GED = General Educational Development, PIR = poverty impact ratio, PIV = pan-immune-inflammatory value.

**Figure 5.**
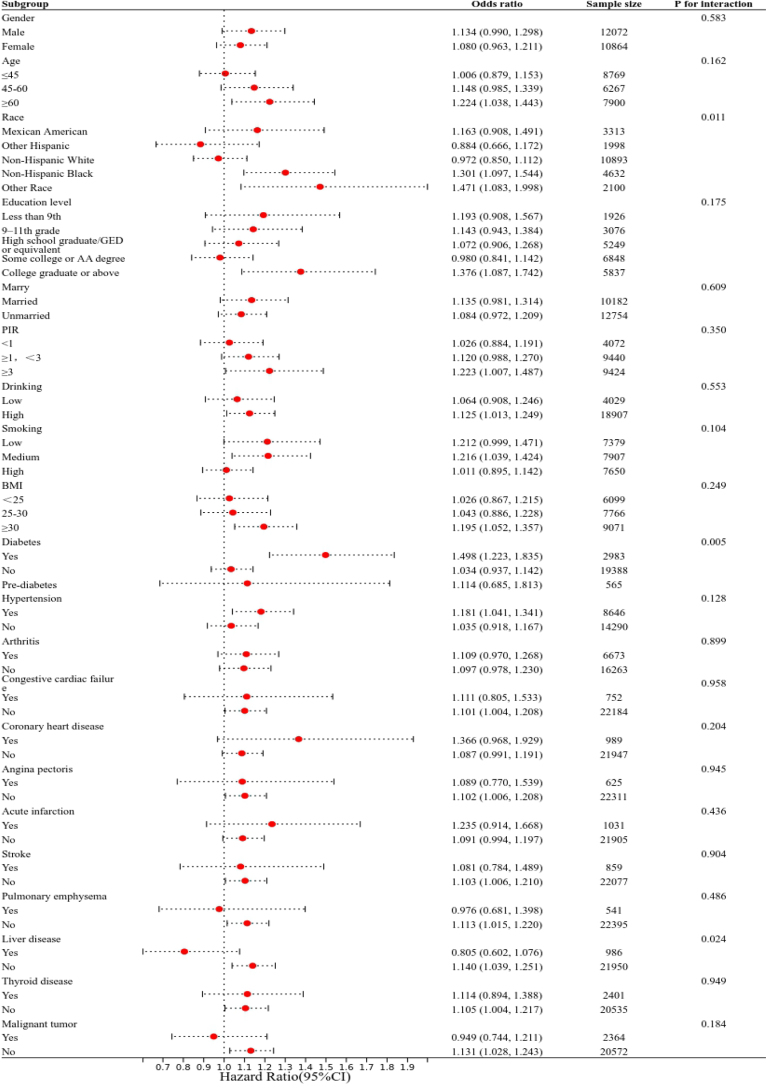
Subgroup analysis of the association between Ln(SIRI) and depression. Models adjusted for age, sex, race, education, marital status, PIR, BMI, smoking, and alcohol (stratification variables excluded). Estimates are survey-weighted. BMI = body mass index, CI = confidence interval, GED = General Educational Development, PIR = poverty impact ratio, SIRI = systemic inflammatory response index.

Subgroup analyses were performed to examine the relationships between Ln(PIV), Ln(SII), and Ln(SIRI) and depression across the demographic and clinical subgroups. For Ln(PIV), ethnicity, smoking status, diabetes, and liver disease were identified as potential effect modifiers (*P* < .05). No significant differences were observed among the other subgroups. A statistically significant interaction between age and smoking status was observed in Ln(SII) analysis (*P* < .05). Subgroup analysis for Ln(SIRI) revealed largely consistent results, with ethnicity, diabetes, and liver disease emerging as modifiers (*P* < .05). These findings suggest that certain populations are more vulnerable to the effects of systemic inflammation on depression. However, these interaction effects should be regarded as exploratory findings, given the number of subgroup comparisons. Therefore, the results should be interpreted cautiously (Figs. 3–5).

## 4. Discussion

Among the 22,936 participants included in this study, those diagnosed with depression exhibited poor health status and socioeconomic conditions across several dimensions, reflecting the complex interaction between socioeconomic factors, lifestyle, and mental health. This study found significant associations between 3 inflammatory markers – SII, PIV, and SIRI – and depression, with elevated levels of these markers correlating with notably higher odds of depression. Smooth curve fitting and threshold effect analysis further revealed a nonlinear “J-curve” relationship between inflammation markers and depression, showing that the impact of inflammation on the odds of prevalent depression increases significantly beyond certain thresholds. Furthermore, stratified analyses revealed that clinical factors, such as smoking, diabetes, and liver disease, modified the strength of the association between these markers and depression, underscoring the importance of interindividual variability in the inflammation–depression relationship. Accordingly, the SII, PIV, and SIRI may be useful cross-sectional correlates of depressive status. Determining their longitudinal trajectories and clinical applicability, including any prognostic or therapeutic relevance, requires prospective cohort studies and interventional trials.

The connection between immune-related inflammation and depression offers new perspectives on the intricate mechanisms driving this condition. SII includes neutrophils, platelets, and lymphocytes. Neutrophils reflect an acute inflammatory response, platelets amplify inflammation through the release of mediators, and lymphocytes indicate an immunosuppressive state. As an indicator of the dynamic interplay between systemic inflammation and immune-inflammatory responses, an elevated SII in patients with depression may result from hyperactivation of neutrophils and suppression of lymphocyte function. Pro-inflammatory factors such as IL-1β and IL-6, released by neutrophils, can cross the brain through peripheral signaling, activate microglia, and trigger neuroinflammation.^[16,17]^ In an extensive cross-sectional analysis involving 42,133 individuals from 2005 to 2020, a 1-unit increment in Ln(SII) was linked to 18% higher odds of prevalent depression.^[18]^ Additionally, SII demonstrated a higher area under the curve for predicting poststroke depression, proving its potential as a reliable marker of inflammation.^[19]^ SIRI, which modifies the SII by excluding platelets and adding monocytes, strengthens the assessment of innate immune activation, particularly microglial activation, while reducing the confounding effect of coagulation.^[20]^ By combining elements of both innate and adaptive immunity, the SIRI provides a broader view of the body’s inflammatory condition. Elevated SIRI in patients with depression may correlate with microglial M1-type polarization, and factors such as IL-6 secreted by monocytes may further exacerbate peripheral inflammation, influencing neurotransmitter metabolism.^[16]^ In a study comprising 2514 adults with depression and 26,487 without depression, a 1-unit increase in SIRI corresponded to a 6% increased likelihood of depression onset.^[16]^ Moreover, SIRI has been shown to independently predict the odds of prevalent poststroke depression and poor treatment responses in patients with refractory bipolar depression.^[21,22]^ PIV, which reintroduces platelets alongside SIRI, combines neutrophils, monocytes, and platelets, thus capturing the broader “immune-coagulation-inflammation axis.” Although direct studies on PIV and depression are scarce, elevated composite parameters such as the neutrophil/lymphocyte and platelet/lymphocyte ratios have been linked to the severity of depression.^[23]^ PIV has also been associated with neurological conditions such as stroke and cognitive disorders, suggesting a role of microglial activation and astrocyte involvement in persistent inflammatory states.^[9,24]^

The link between depression and immune inflammation involves both peripheral immune activation and transmission of inflammatory signals to the brain. Individuals with depression often show elevated levels of pro-inflammatory cytokines, such as IL-6 and IL-1β, triggered by stress, infections, or autoimmune conditions. These cytokines activate the hypothalamic–pituitary–adrenal axis, increasing cortisol levels and disrupting the immune balance, thereby promoting systemic inflammation.^[25]^ Altered gut microbiota may further contribute to inflammation through the gut–brain axis, particularly via increased intestinal permeability.^[26]^ These peripheral signals can cross the blood–brain barrier through active transport, damaged regions, or neural pathways,^[27]^ initiating microglial and astrocyte activation and creating a pro-inflammatory environment within the brain. Chronic microglial activation amplifies neuroinflammation and is often associated with stress- and oxidation-induced barrier dysfunction.^[28]^ Inflammation also disrupts neurotransmitter systems. It reduces serotonin synthesis by diverting tryptophan metabolism through the indoleamine 2,3-dioxygenase pathway and affects the glutamate balance.^[29]^ Moreover, it suppresses brain-derived neurotrophic factor, impairing neurogenesis and plasticity in the hippocampus and prefrontal cortex.^[30]^ Finally, inflammation interferes with mood regulation circuits, such as the amygdala–prefrontal network, intensifying negative emotion processing and stress reactivity.^[31]^ Future research should further investigate the dynamics of inflammation at different stages of depression and assess the long-term effectiveness of anti-inflammatory strategies in precision medicine for depression treatment.

This research uncovered a notable link between increased values of the inflammatory markers Ln(PIV), Ln(SII), and Ln(SIRI) and the onset of depression. The natural logarithmic transformation of these indices standardizes their distributions, enhancing the robustness of statistical comparisons. Subgroup analyses indicated that factors such as age, race, smoking, liver disease, and diabetes may serve as important modifiers of the relationship between inflammatory indices and depression. For Ln(PIV), race, smoking status, diabetes, and liver disease were significantly associated with depression prevalence, consistent with prior studies showing that chronic inflammatory conditions (e.g., smoking, diabetes, and liver disease) can worsen depressive symptoms by amplifying systemic inflammation.^[32–34]^The observed racial differences in inflammatory responses may be attributable to a complex interplay of factors, including genetic predisposition, culturally influenced health behaviors, and socioeconomic disparities.^[35]^ Age and smoking showed significant interactions for Ln(SII), suggesting that long-term smoking may exacerbate the odds of depression, particularly in older individuals, which is consistent with studies linking chronic inflammation and psychiatric outcomes related to prolonged smoking.^[36]^ For Ln(SIRI), race, diabetes, and liver disease were significant moderators, further supporting the hypothesis that these conditions influence the relationship between inflammation and depression. These results highlight the importance of tailored depression management for patients with particular characteristics (or associated factors) such as age, ethnicity, smoking, diabetes, and liver disease. However, given the multiple primary comparisons across the 3 biomarkers and their different parameterizations, these findings should be regarded as exploratory and interpreted with caution. We emphasized effect sizes and 95% CIs rather than relying solely on *P* values to convey the strength and direction of the associations. Future research should incorporate appropriate multiplicity control and investigate underlying inflammatory mechanisms to confirm these associations in larger and more diverse populations. To ensure clarity, our models were designed for association rather than prediction; consequently, diagnostic metrics, such as calibration or discrimination, were not the focus of this study. Future prognostic modeling efforts should further evaluate these aspects.

While prior studies have examined the associations between environmental exposure, diet, lifestyle, and depression, this study provides a more systematic evaluation of inflammation-related markers, such as SII, PIV, and SIRI, offering new perspectives for optimizing depression treatment strategies.^[37–39]^ Effectively managing and mitigating inflammatory conditions is essential to prevent and intervene early in depression. Research suggests that long-term antidepressant use may help alleviate depression by reducing inflammation, such as by inhibiting lipopolysaccharide-induced depressive-like behavior.^[40]^ A meta-analysis of 48 studies confirmed a significant antidepressant effect of anti-inflammatory drugs in major depressive disorder (OR = 2.04, *P* = .0002), particularly in adjunctive and non-refractory patients.^[41]^ These findings highlight the therapeutic potential of targeting inflammation in depression and support the use of SII, PIV, and SIRI as promising biomarkers for identifying the underlying inflammatory mechanisms. Integrating these markers into clinical assessments may aid in early identification and guide the development of personalized anti-inflammatory interventions.

## 5. Study strengths and limitations

This investigation utilized NHANES, a comprehensive, nationally representative dataset encompassing 22,936 participants, providing a robust and generalizable view of the connection between inflammatory markers and depression among U.S. adults. This represents an inaugural effort to methodically examine the associations of SII, PIV, and SIRI with depression, highlighting the possible influence of inflammation on the emergence of depressive symptoms. Furthermore, this research uncovered a “J-shaped” nonlinear association and threshold effects between inflammation markers and depression, offering a fresh perspective for exploring the intricate connections between inflammation and mental well-being. However, this study had several limitations. First, the cross-sectional design precludes causal inference, and elevated inflammatory markers may represent consequences rather than causes of depression. Second, depression was assessed using self-reported screening tools (e.g., the PHQ-9), which are subject to recall bias and diagnostic variability. Although a PHQ-9 cutoff of ≥10 is clinically meaningful, dichotomization may reduce information compared to continuous or ordinal modeling. Third, SII, PIV, and SIRI are highly correlated. To avoid multicollinearity, we analyzed them in separate models, which may not fully disentangle their overlapping contributions. Fourth, smoking exposure was assessed using serum cotinine alone, without reconciliation with self-reports, which may be considered a limitation. Fifth, the restricted cubic spline analyses suggested J-shaped associations and approximate inflection points. However, these thresholds are exploratory and data-driven, without internal validation or sensitivity to knot placement, and should therefore be interpreted with caution. Sixth, residual confounding remains possible. The NHANES lacks consistent data on pregnancy, autoimmune conditions, and certain medications (e.g., steroids, NSAIDs, and psychotropics). Additionally, consistent exclusion of participants with acute inflammation (e.g., CRP > 10 mg/L) across the entire study period was not feasible because of the lack of CRP data in the 2011 to 2014 survey cycles. However, sensitivity analyses indicated that unmeasured confounding would need to be substantial to negate the observed associations; thus, while residual confounding cannot be fully excluded, it is unlikely to invalidate the main findings.^[42]^ Thus, a cautious interpretation remains necessary. Seventh, although we applied sensitivity analyses, survey weights, and standardized CBC assays across cycles, residual temporal heterogeneity and model instability cannot be entirely excluded. Eighth, complete-case analysis may introduce bias, but missingness was negligible and the excluded participants were generally comparable, making substantial bias unlikely. Finally, as the NHANES primarily reflects the U.S. population, the generalizability of our findings to other settings is limited. Future longitudinal and diverse cohorts are needed to confirm the predictive validity.

## 6. Conclusion

Through an analysis of data from the NHANES spanning 2005 to 2018, this study observed a significant curvilinear J-shaped dose-response relationship between 3 systemic inflammatory biomarkers (SIRI, SII, and PIV) and depressive symptoms. This finding suggests that the body’s inflammatory state may act as a key modulator of the pathological mechanisms of depression, indicating that interventions targeting inflammatory pathways could represent a novel public health strategy to alleviate the burden of mental health disorders. This study underscores the need for an in-depth exploration of neuroimmune interaction mechanisms and calls for clinical translational research to validate the potential utility of anti-inflammatory therapies in the management of affective disorders.

## Acknowledgments

We acknowledge the National Center for Health Statistics (NCHS) for providing access to the NHANES data.

## Author contributions

**Conceptualization:** Guanwen Zhu.

**Data curation:** Jiawen Liu.

**Formal analysis:** Guanwen Zhu, Jiawen Liu.

**Funding acquisition:** Sijia Wang.

**Investigation:** Jiawen Liu.

**Methodology:** Guanwen Zhu.

**Project administration:** Sijia Wang.

**Supervision:** Long Wang.

**Validation:** Jiawen Liu.

**Writing – original draft:** Guanwen Zhu.

**Writing – review & editing:** Guanwen Zhu, Sijia Wang, Long Wang.


