# Supplementary material for: Systemic inflammation and mental health: A J-shaped dose-response pattern in depression based on NHANES 2005 to 2018
Source: Medicine (Baltimore). 2026 Jul 3;105(27):e49348. doi: 10.1097/MD.0000000000049348 (PMC13336952; doi:10.1097/MD.0000000000049348)
Supplement: Supplementary file 1 [file medi-105-e49348-s001.docx]

**Supplemental File:** R Code for Analysis of Systemic Inflammation Markers and Depression in NHANES 2005-2018

suppressPackageStartupMessages({

  library(nhanesA)

  library(survey)

  library(dplyr)

  library(tidyr)

  library(stringr)

  library(purrr)

  library(ggplot2)

  library(broom)

  library(data.table)

  library(splines)

})

# ------------------------------------------------------------

# 0. Configuration

# ------------------------------------------------------------

# NHANES cycles used: 2005–2018 (D–J)

cycle_suffixes <- c("D","E","F","G","H","I","J")

# Output directory (current working directory). Modify if needed.

out_dir <- getwd()

# Helper to write CSV with standard options

write_csv2 <- function(df, path) {

  readr::write_csv(df, path, na = "")

}

# ------------------------------------------------------------

# 1. Defensive data-access utilities

# ------------------------------------------------------------

empty_df <- function(cols = c(SEQN = integer())) {

  as.data.frame(lapply(cols, function(proto) proto[0]))

}

REQUIRED_MIN_COLS <- c(SEQN = integer())

safe_pull_cycle <- function(table, sfx,

                            required_cols = REQUIRED_MIN_COLS,

                            add_cycle = TRUE) {

  nm <- paste0(table, "_", sfx)

  res <- tryCatch(nhanesA::nhanes(nm), error = function(e) empty_df(required_cols))

  if (!inherits(res, "data.frame")) res <- as.data.frame(res)

  if (!"SEQN" %in% names(res)) res$SEQN <- NA_integer_

  res$SEQN <- as.integer(res$SEQN)

  if (add_cycle && !"SURVEY_CYCLE" %in% names(res)) res$SURVEY_CYCLE <- sfx

  miss <- setdiff(names(required_cols <- names(required_cols)), names(res))

  if (length(miss)) for (m in miss) res[[m]] <- NA

  res

}

harmonize_race <- function(demo){

  if ("RIDRETH3" %in% names(demo) && "RIDRETH1" %in% names(demo)) {

    demo$RACE_ETH <- dplyr::coalesce(demo$RIDRETH3, demo$RIDRETH1)

  } else if ("RIDRETH3" %in% names(demo)) demo$RACE_ETH <- demo$RIDRETH3

  else if ("RIDRETH1" %in% names(demo))  demo$RACE_ETH <- demo$RIDRETH1

  else demo$RACE_ETH <- NA

  demo

}

find_cbc_vars <- function(cbc_df){

  nms <- names(cbc_df)

  candidates <- list(

    lymph_abs = c("LBXLYMNO","LBXLYM","LBXLYMNE","LBDLYMNO","LBDLYM"),

    neut_abs  = c("LBXNEU","LBXNEUN","LBXNEUO","LBXNEU3","LBXNE","LBDNEU"),

    mono_abs  = c("LBXMNO","LBXMONO","LBXMON","LBDMONO","LBXMO"),

    platelets = c("LBXPLTSI","LBXPLTS","LBXPLT")

  )

  out <- lapply(candidates, function(v) { hit <- v[v %in% nms]; if (length(hit)) hit[1] else NA_character_ })

  out

}

compute_phq9 <- function(dpq){

  items <- sprintf("DPQ%03d", seq(10, 90, by = 10))

  have <- items[items %in% names(dpq)]

  for (v in have) dpq[[v]] <- ifelse(dpq[[v]] %in% c(7,9), NA, dpq[[v]])

  dpq$PHQ9_SUM <- rowSums(dpq[, have, drop = FALSE], na.rm = FALSE)

  dpq$DEPRESSED <- ifelse(dpq$PHQ9_SUM >= 10, 1, 0)

  dpq

}

recode_comorbs <- function(mcq, diq, bpq){

  out <- data.frame(SEQN = dplyr::coalesce(mcq$SEQN, diq$SEQN, bpq$SEQN))

  out$STROKE   <- if ("MCQ160F" %in% names(mcq)) ifelse(mcq$MCQ160F==1,1, ifelse(mcq$MCQ160F %in% c(2,3),0,NA)) else NA

  out$CHF      <- if ("MCQ160B" %in% names(mcq)) ifelse(mcq$MCQ160B==1,1, ifelse(mcq$MCQ160B %in% c(2,3),0,NA)) else NA

  out$ARTH     <- if ("MCQ160A" %in% names(mcq)) ifelse(mcq$MCQ160A==1,1, ifelse(mcq$MCQ160A %in% c(2,3),0,NA)) else NA

  out$DIABETES <- if ("DIQ010"  %in% names(diq)) ifelse(diq$DIQ010==1, 1, ifelse(diq$DIQ010 %in% c(2,3),0,NA)) else NA

  out$HTN      <- if ("BPQ020"  %in% names(bpq)) ifelse(bpq$BPQ020==1, 1, ifelse(bpq$BPQ020 %in% c(2,3),0,NA)) else NA

  out$LIVER    <- if ("MCQ160L" %in% names(mcq)) ifelse(mcq$MCQ160L==1,1, ifelse(mcq$MCQ160L %in% c(2,3),0,NA)) else NA

  out

}

derive_alcohol <- function(alq){

  out <- data.frame(SEQN = alq$SEQN)

  drink_now <- if ("ALQ101" %in% names(alq)) ifelse(alq$ALQ101 %in% c(1), 1, 0) else NA

  days12 <- if ("ALQ120Q" %in% names(alq)) alq$ALQ120Q else NA

  freq_cat <- ifelse(is.na(days12) | (drink_now == 0), 0,

                ifelse(days12 <= 12, 1,

                ifelse(days12 <= 48, 2,

                ifelse(days12 <= 156, 3, 4))))

  out$ALC_FREQ <- freq_cat

  out

}

cotinine_tertiles <- function(cot){

  out <- data.frame(SEQN = cot$SEQN)

  if (!("LBXCOT" %in% names(cot))) { out$COT_TERT <- NA_integer_; return(out) }

  qs <- quantile(cot$LBXCOT, probs = c(1/3, 2/3), na.rm = TRUE, type = 7)

  out$COT_TERT <- cut(cot$LBXCOT, breaks = c(-Inf, qs[1], qs[2], Inf), labels = c(1,2,3), right = TRUE)

  out$COT_TERT <- as.integer(as.character(out$COT_TERT))

  out

}

# ------------------------------------------------------------

# 2. Cycle-wise pulls for DEMO, CBC, DPQ (for flow counts)

# ------------------------------------------------------------

get_demo_all <- function(){

  demos <- lapply(cycle_suffixes, function(sfx){

    d <- safe_pull_cycle("DEMO", sfx)

    if (nrow(d) == 0) return(d)

    d <- harmonize_race(d)

    keep <- intersect(names(d), c("SEQN","RIDAGEYR","WTMEC2YR","SDMVPSU","SDMVSTRA",

                                  "RIAGENDR","RACE_ETH","DMDEDUC2","INDFMPIR","DMDMARTL"))

    d[, keep, drop = FALSE]

  })

  data.table::rbindlist(demos, use_names = TRUE, fill = TRUE)

}

get_cbc_all <- function(){

  cbc <- lapply(cycle_suffixes, function(sfx){

    c <- safe_pull_cycle("CBC", sfx)

    if (nrow(c) == 0) return(c)

    vs <- find_cbc_vars(c)

    keep <- unique(na.omit(c("SEQN", vs$platelets, vs$neut_abs, vs$lymph_abs, vs$mono_abs)))

    if (!length(keep)) return(empty_df(c(SEQN=integer())))

    c <- c[, keep, drop = FALSE]

    names(c)[names(c)==vs$platelets] <- "PLATELETS"

    names(c)[names(c)==vs$neut_abs ] <- "NEU_ABS"

    names(c)[names(c)==vs$lymph_abs] <- "LYM_ABS"

    names(c)[names(c)==vs$mono_abs ] <- "MONO_ABS"

    c

  })

  data.table::rbindlist(cbc, use_names = TRUE, fill = TRUE)

}

get_dpq_all <- function(){

  dpq <- lapply(cycle_suffixes, function(sfx){

    d <- safe_pull_cycle("DPQ", sfx)

    if (nrow(d) == 0) return(d)

    compute_phq9(d)

  })

  data.table::rbindlist(dpq, use_names = TRUE, fill = TRUE)

}

message("Pulling DEMO/CBC/DPQ for flow counts...")

demo_all <- get_demo_all()

cbc_all  <- get_cbc_all()

dpq_all  <- get_dpq_all()

N0 <- nrow(demo_all)

demo_adult <- dplyr::filter(demo_all, RIDAGEYR >= 18)

N_adult <- nrow(demo_adult)

adult_cbc <- merge(demo_adult, cbc_all, by="SEQN", all.x = TRUE)

no_cbc <- with(adult_cbc, is.na(PLATELETS) | is.na(NEU_ABS) | is.na(LYM_ABS) | is.na(MONO_ABS))

N_no_cbc <- sum(no_cbc, na.rm=TRUE)

adult_cbc_ok <- adult_cbc[!no_cbc, ]

adult_cbc_dpq <- merge(adult_cbc_ok, dpq_all[, c("SEQN","DEPRESSED")], by="SEQN", all.x=TRUE)

N_no_depr <- sum(is.na(adult_cbc_dpq$DEPRESSED))

adult_cbc_dpq_ok <- adult_cbc_dpq[!is.na(adult_cbc_dpq$DEPRESSED), ]

# Cumulative consistency checks

stopifnot(N0 == nrow(demo_all))

stopifnot(N_adult == nrow(demo_adult))

stopifnot(N_no_cbc + nrow(adult_cbc_ok) == nrow(adult_cbc))

stopifnot(N_no_depr + nrow(adult_cbc_dpq_ok) == nrow(adult_cbc_ok))

flow_counts <- data.frame(

  Step = c("All participants","Age >= 18","Missing CBC (any)","Missing PHQ-9","Final analytic N"),

  N = c(N0, N_adult, N_no_cbc, N_no_depr, nrow(adult_cbc_dpq_ok))

)

readr::write_csv(flow_counts, file.path(out_dir, "Flow_Counts.csv"))

# ------------------------------------------------------------

# 3. Full per-cycle merge for analytic dataset

# ------------------------------------------------------------

merge_cycle <- function(sfx){

  demo <- safe_pull_cycle("DEMO", sfx)

  dpq  <- safe_pull_cycle("DPQ",  sfx)

  bmx  <- safe_pull_cycle("BMX",  sfx)

  cbc  <- safe_pull_cycle("CBC",  sfx)

  cot  <- safe_pull_cycle("COT",  sfx)

  alq  <- safe_pull_cycle("ALQ",  sfx)

  bpq  <- safe_pull_cycle("BPQ",  sfx)

  diq  <- safe_pull_cycle("DIQ",  sfx)

  mcq  <- safe_pull_cycle("MCQ",  sfx)

  if (nrow(demo)) demo <- harmonize_race(demo)

  if (nrow(dpq))  dpq  <- compute_phq9(dpq)

  if (nrow(bmx) && "BMXBMI" %in% names(bmx)) bmx <- bmx[, c("SEQN","BMXBMI")]

  if (nrow(cbc)) {

    vs <- find_cbc_vars(cbc)

    keep <- unique(na.omit(unlist(c("SEQN", vs$platelets, vs$neut_abs, vs$lymph_abs, vs$mono_abs))))

    cbc <- cbc[, keep, drop=FALSE]

    names(cbc)[names(cbc)==vs$platelets] <- "PLATELETS"

    names(cbc)[names(cbc)==vs$neut_abs ] <- "NEU_ABS"

    names(cbc)[names(cbc)==vs$lymph_abs] <- "LYM_ABS"

    names(cbc)[names(cbc)==vs$mono_abs ] <- "MONO_ABS"

  }

  if (nrow(cot)) cot <- cotinine_tertiles(cot)

  if (nrow(alq)) alq <- derive_alcohol(alq)

  if (nrow(mcq) | nrow(diq) | nrow(bpq)) {

    if (!nrow(mcq)) mcq <- data.frame(SEQN = integer())

    if (!nrow(diq)) diq <- data.frame(SEQN = integer())

    if (!nrow(bpq)) bpq <- data.frame(SEQN = integer())

    tmp <- Reduce(function(a,b) merge(a,b, by="SEQN", all=TRUE), list(mcq, diq, bpq))

    comorb <- recode_comorbs(tmp, tmp, tmp)

  } else comorb <- NULL

  if (nrow(demo)) {

    keep_demo <- c("SEQN","WTMEC2YR","SDMVPSU","SDMVSTRA","RIDAGEYR","RIAGENDR","RACE_ETH","DMDEDUC2","INDFMPIR","DMDMARTL")

    demo <- demo[, intersect(keep_demo, names(demo)), drop = FALSE]

  }

  dfs <- list(demo, dpq, bmx, cbc, cot, alq, comorb)

  dfs <- dfs[ vapply(dfs, function(z) !is.null(z) && nrow(z)>0, logical(1)) ]

  if (!length(dfs)) return(NULL)

  Reduce(function(a,b) merge(a,b, by="SEQN", all=FALSE), dfs)

}

message("Merging cycles for analytic dataset...")

dt_list <- lapply(cycle_suffixes, merge_cycle)

dt_list <- dt_list[!sapply(dt_list, is.null)]

included_cycles <- length(dt_list)

nh <- data.table::rbindlist(dt_list, use_names = TRUE, fill = TRUE)

# ------------------------------------------------------------

# 4. Analytic sample filters & biomarker indices

# ------------------------------------------------------------

nh <- nh %>% filter(RIDAGEYR >= 18)

nh <- nh %>% filter(!is.na(PLATELETS) & !is.na(NEU_ABS) & !is.na(LYM_ABS) & !is.na(MONO_ABS))

nh <- nh %>% filter(!is.na(DEPRESSED))

# Physiological plausibility filter to avoid exploding ratios (zero/negative)

nh <- nh %>% filter(PLATELETS > 0, NEU_ABS > 0, LYM_ABS > 0, MONO_ABS > 0)

nh$SII  <- (nh$PLATELETS * nh$NEU_ABS) / nh$LYM_ABS

nh$PIV  <- (nh$PLATELETS * nh$NEU_ABS * nh$MONO_ABS) / nh$LYM_ABS

nh$SIRI <- (nh$NEU_ABS * nh$MONO_ABS) / nh$LYM_ABS

winsor <- function(x, p=0.001){

  qs <- quantile(x, c(p, 1-p), na.rm = TRUE)

  pmax(pmin(x, qs[2]), qs[1])

}

nh$lnSII  <- log(winsor(nh$SII))

nh$lnPIV  <- log(winsor(nh$PIV))

nh$lnSIRI <- log(winsor(nh$SIRI))

# ------------------------------------------------------------

# 5. Covariate missingness & complete-case

# ------------------------------------------------------------

covars_needed <- c("RIDAGEYR","RIAGENDR","RACE_ETH","DMDEDUC2","DMDMARTL","INDFMPIR","BMXBMI","COT_TERT","ALC_FREQ")

miss_tab <- sapply(nh[, covars_needed, drop=FALSE], function(x) sum(!is.finite(x) | is.na(x)))

cov_miss <- data.frame(variable=names(miss_tab), n_missing=as.integer(miss_tab))

readr::write_csv(cov_miss, file.path(out_dir, "Covariate_Missing_Counts.csv"))

N_missing_cov <- sum(!complete.cases(nh[, covars_needed, drop=FALSE]))

nh <- nh %>% filter(complete.cases(across(all_of(covars_needed))))

# ------------------------------------------------------------

# 6. Pooled weights (ACTUAL cycles) & survey design

# ------------------------------------------------------------

nh$WTMEC_POOL <- nh$WTMEC2YR / included_cycles

# If cross-cycle duplicate STRATA/PSU codes are detected, enable the following:

# nh$cycle_id <- nh$SURVEY_CYCLE

# nh$SDMVSTRA <- interaction(nh$cycle_id, nh$SDMVSTRA, drop=TRUE)

# nh$SDMVPSU  <- interaction(nh$cycle_id, nh$SDMVPSU,  drop=TRUE)

des <- svydesign(id=~SDMVPSU, strata=~SDMVSTRA, weights=~WTMEC_POOL, nest=TRUE, data=nh)

# ------------------------------------------------------------

# 7. Factor levels aligned in data layer (no factor() in formulas)

# ------------------------------------------------------------

to_factor <- c("RIAGENDR","RACE_ETH","DMDEDUC2","DMDMARTL","COT_TERT","ALC_FREQ")

for (v in to_factor) if (v %in% names(nh)) nh[[v]] <- factor(nh[[v]])

des <- update(des, data = nh)

cov_m2 <- c("RIDAGEYR","RIAGENDR","RACE_ETH")

cov_m3 <- c(cov_m2,"DMDEDUC2","DMDMARTL","INDFMPIR","BMXBMI","COT_TERT","ALC_FREQ")

# ------------------------------------------------------------

# 8. RCS (nonlinearity tests) & curve export

# ------------------------------------------------------------

rcs_basis_fixed <- function(x){

  probs <- c(0.05, 0.35, 0.65, 0.95)   # pre-specified percentiles

  knots <- as.numeric(quantile(x, probs = probs, na.rm = TRUE))

  internal <- knots[2:3]

  basis <- splines::ns(x, knots = internal, Boundary.knots = range(x, na.rm = TRUE))

  attr(basis, "knots_all") <- knots

  basis

}

models <- list(

  list(name="lnSII",  x="lnSII",  label="ln(SII)"),

  list(name="lnPIV",  x="lnPIV",  label="ln(PIV)"),

  list(name="lnSIRI", x="lnSIRI", label="ln(SIRI)")

)

rcs_tests <- list()

spline_plot_data <- list()

levs <- lapply(to_factor, function(v) levels(nh[[v]])); names(levs) <- to_factor

for (m in models) {

  x <- m$x

  basis <- rcs_basis_fixed(nh[[x]])

  bnames <- paste0(x, "_rcs", seq_len(ncol(basis)))

  nh_rcs <- cbind(nh, as.data.frame(basis)); names(nh_rcs)[(ncol(nh_rcs)-ncol(basis)+1):ncol(nh_rcs)] <- bnames

  des_rcs <- svydesign(id=~SDMVPSU, strata=~SDMVSTRA, weights=~WTMEC_POOL, nest=TRUE, data=nh_rcs)

  rhs <- paste(c(bnames, cov_m3), collapse = " + ")

  fit_rcs <- svyglm(as.formula(paste0("DEPRESSED ~ ", rhs)), design = des_rcs, family = quasibinomial())

  fit_lin <- svyglm(as.formula(paste0("DEPRESSED ~ ", x, " + ", paste(cov_m3, collapse = " + "))), design = des, family = quasibinomial())

  L <- anova(fit_lin, fit_rcs)

  p_nl <- tryCatch(L[2,"Pr(>F)"], error=function(e) NA_real_)

  rcs_tests[[m$name]] <- data.frame(Biomarker=m$name, P_Nonlinearity = p_nl)

  grid <- data.frame(xseq = seq(quantile(nh[[x]], 0.01, na.rm=TRUE), quantile(nh[[x]], 0.99, na.rm=TRUE), length.out = 200))

  basis_grid <- predict(basis, newx = grid$xseq)

  colnames(basis_grid) <- bnames

  newdat <- cbind(grid, as.data.frame(basis_grid))

  ref <- nh %>% dplyr::summarise(

    RIDAGEYR = mean(RIDAGEYR, na.rm = TRUE),

    INDFMPIR = mean(INDFMPIR, na.rm = TRUE),

    BMXBMI   = mean(BMXBMI,   na.rm = TRUE),

    RIAGENDR = names(sort(table(RIAGENDR), decreasing=TRUE))[1],

    RACE_ETH = names(sort(table(RACE_ETH), decreasing=TRUE))[1],

    DMDEDUC2 = names(sort(table(DMDEDUC2), decreasing=TRUE))[1],

    DMDMARTL = names(sort(table(DMDMARTL), decreasing=TRUE))[1],

    COT_TERT = names(sort(table(COT_TERT), decreasing=TRUE))[1],

    ALC_FREQ = names(sort(table(ALC_FREQ), decreasing=TRUE))[1]

  )

  newdat$RIDAGEYR <- ref$RIDAGEYR; newdat$INDFMPIR <- ref$INDFMPIR; newdat$BMXBMI <- ref$BMXBMI

  newdat$RIAGENDR <- factor(ref$RIAGENDR, levels = levs$RIAGENDR)

  newdat$RACE_ETH <- factor(ref$RACE_ETH, levels = levs$RACE_ETH)

  newdat$DMDEDUC2 <- factor(ref$DMDEDUC2, levels = levs$DMDEDUC2)

  newdat$DMDMARTL <- factor(ref$DMDMARTL, levels = levs$DMDMARTL)

  newdat$COT_TERT <- factor(ref$COT_TERT, levels = levs$COT_TERT)

  newdat$ALC_FREQ <- factor(ref$ALC_FREQ, levels = levs$ALC_FREQ)

  preds <- predict(fit_rcs, newdata=newdat, type="link", se.fit=TRUE)

  newdat$OR      <- exp(preds$fit - preds$fit[1])

  newdat$OR_low  <- exp(preds$fit - 1.96*preds$se.fit - preds$fit[1])

  newdat$OR_high <- exp(preds$fit + 1.96*preds$se.fit - preds$fit[1])

  newdat$Biomarker <- m$label; newdat$xval <- newdat$xseq

  spline_plot_data[[m$name]] <- newdat

  # Export curve data (per biomarker)

  readr::write_csv(newdat, file.path(out_dir, paste0("Spline_Curve_", m$name, ".csv")))

  # Plot and export

  p <- ggplot(newdat, aes(x = xval, y = OR)) +

    geom_line() +

    geom_ribbon(aes(ymin = OR_low, ymax = OR_high), alpha = 0.2, linewidth = 0) +

    labs(x = m$label, y = "Odds Ratio (vs. left boundary)", title = paste0("RCS Curve: ", m$label)) +

    theme_bw(base_size = 12)

  ggsave(filename = file.path(out_dir, paste0("Figure_RCS_", m$name, ".png")), plot = p, width = 6, height = 4, dpi = 300)

}

rcs_out <- bind_rows(rcs_tests)

readr::write_csv(rcs_out, file.path(out_dir, "Nonlinearity_Tests.csv"))

# ------------------------------------------------------------

# 9. Exploratory threshold scan + Davies test (approximate)

# ------------------------------------------------------------

fit_threshold_scan <- function(design, y, x, covars, grid=NULL){

  df <- model.frame(design); xv <- df[[x]]

  if (is.null(grid)) grid <- unique(as.numeric(quantile(xv, probs = seq(0.1, 0.9, by = 0.01), na.rm = TRUE)))

  best <- list(aic=Inf, bp=NA, fit=NULL)

  for (bp in grid) {

    df$seg1 <- pmin(xv, bp); df$seg2 <- pmax(xv - bp, 0)

    fml <- as.formula(paste(y, "~ seg1 + seg2 +", paste(covars, collapse = " + ")))

    des2 <- update(design, ~ .); des2$variables <- df

    fit <- suppressWarnings(svyglm(fml, design = des2, family = quasibinomial()))

    aic <- AIC(fit)

    if (is.finite(aic) && aic < best$aic) best <- list(aic=aic, bp=bp, fit=fit)

  }

  best

}

th_scan <- lapply(models, function(m) fit_threshold_scan(des, y="DEPRESSED", x=m$x, covars=cov_m3, grid=NULL))

names(th_scan) <- sapply(models, `[[`, "name")

th_out <- purrr::map_df(names(th_scan), function(nm){

  x <- th_scan[[nm]]

  data.frame(Biomarker = nm, Best_AIC = x$aic, Breakpoint = x$bp)

})

readr::write_csv(th_out, file.path(out_dir, "Threshold_Scan_Summary.csv"))

davies_tbl <- NULL

if (requireNamespace("segmented", quietly = TRUE)) {

  suppressPackageStartupMessages(library(segmented))

  df_mf <- model.frame(des)

  davies_tbl <- purrr::map_df(models, function(m){

    f_glm <- as.formula(paste("DEPRESSED ~", paste(c(m$x, cov_m3), collapse=" + ")))

    glm_fit <- glm(f_glm, data = df_mf, family = binomial())

    seg_fit <- try(segmented::segmented(glm_fit, seg.Z = as.formula(paste("~", m$x))), silent = TRUE)

    pv <- if (inherits(seg_fit, "segmented")) summary(seg_fit)$davies.test$p.value else NA_real_

    data.frame(Biomarker = m$name, Davies_p = pv)

  })

  readr::write_csv(davies_tbl, file.path(out_dir, "Davies_Test_pvalues.csv"))

}

# ------------------------------------------------------------

# 10. Session info (for strict reproducibility)

# ------------------------------------------------------------

sink(file.path(out_dir, "Session_Info.txt"))

sessionInfo()

sink()

message("All outputs written to: ", out_dir)

**Supplementary Note**

All analyses were performed using **R version 4.2.3 (R Foundation for Statistical Computing, Vienna, Austria)** and **EmpowerStats 2.0** under **Windows OS**.
To ensure reproducibility, we also provide the complete output of sessionInfo() (file name: **Session_Info.txt**) in this Supplement. This file details the operating system, R version, and the versions of all loaded R packages.
